# The impact of routine Edmonton Symptom Assessment System (ESAS) use on overall survival in cancer patients: Results of a population‐based retrospective matched cohort analysis

**DOI:** 10.1002/cam4.3374

**Published:** 2020-08-14

**Authors:** Lisa Barbera, Rinku Sutradhar, Hsien Seow, Nicole Mittmann, Doris Howell, Craig C. Earle, Qing Li, Deva Thiruchelvam

**Affiliations:** ^1^ Institute for Clinical Evaluative Sciences Toronto Ontario Canada; ^2^ University of Toronto Toronto Ontario Canada; ^3^ Sunnybrook Research Institute Toronto Ontario Canada; ^4^ Department of Oncology University of Calgary Calgary Alberta Canada; ^5^ Department of Oncology McMaster University Hamilton Ontario Canada; ^6^ University Health Network Princess Margaret Cancer Centre Toronto Ontario Canada

**Keywords:** cohort study, Neoplasms, patient reported outcome measures, propensity score, survival, symptom assessment

## Abstract

**Background:**

The Edmonton Symptom Assessment System (ESAS) is a validated instrument whose use has been standardized in the Ontario cancer system to measure symptoms among ambulatory cancer patients. The objective was to examine the effect of ESAS exposure on overall survival. We hypothesized, a priori, that patients exposed to ESAS would have higher rates of overall survival than those who were not exposed.

**Methods:**

This was a retrospective matched cohort study of adults diagnosed with cancer between 2007 and 2015. Patients were considered exposed if they were screened with ESAS at least once during the study period. Their first ESAS screening date defined the index date. Each exposed patient was matched randomly to a cancer patient without ESAS using a combination of hard matching (4 variables) and propensity score matching (14 variables). Kaplan‐Meier curves and multivariable Cox regression were used to evaluate the impact of ESAS exposure on survival.

**Results:**

There were 128,893 pairs well matched on all baseline characteristics. The probability of survival within the first 5 years was higher among those exposed to ESAS compared to those who were not (81.9% vs. 76.4% at 1 year, 68.3% vs. 66.1% at 3 years, 61.9% vs. 61.4% at 5 years, *P*‐value < .0001). In the multivariable Cox regression model, ESAS was significantly associated with a decreased mortality risk (HR: 0.48, 95% CI: 0.47‐0.49).

**Conclusions:**

Our results show that ESAS exposure is associated with improved survival in cancer patients. This provides real world evidence of the impact of routine symptom assessment in cancer care.

## Graphical abstract

We evaluated the impact of routine symptom screening, using the Edmonton Symptom Assessment System (ESAS), on overall survival in cancer patients. ESAS is used routinely in clinical practice in the cancer system in Ontario, Canada. In this retrospective matched cohort study of 128,893 pairs of cancer patients, those exposed to ESAS during their care had improved survival with an adjusted hazard ratio of 0.49 (95% CI: 0.48‐0.49) when compared to those not exposed. This study provides real world evidence of the impact of ESAS exposure on overall survival which is consistent with findings from randomized trials.

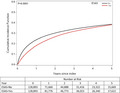

## 1 INTRODUCTION

Routine use of patient reported outcomes (PROs) in routine clinical care is a mechanism to improve the person‐centred nature of care. Several overviews have demonstrated that using PROs routinely in care can improve identification of symptoms, monitoring symptoms over time, quality of life and communication with the team.^1^, ^2^, ^3^, ^4^ Furthermore, emerging results have suggested that routine use of PROs can decrease the use of acute care services such as visits to the emergency department.^5^, ^6^ More enticing are results that their routine use improves survival.^7^, ^8^

In 2007, Cancer Care Ontario implemented a program of routine symptom screening with the Edmonton Symptom Assessment System^9^ (ESAS) for ambulatory oncology patients attending clinics around the province. ESAS assesses 9 common cancer symptoms on a scale of 0 to 10. Most centers have implemented the measure for every visit regardless of cancer type or place in the illness trajectory. Over the past decade, this program has grown and matured.^10^ About 60% of eligible patients are screened^10^, ^11^; 30 000‐40 000 symptoms assessments are collected each month; and, over 5 million symptom records have been captured since the beginning of the program. This makes Ontario's cancer system a unique region to evaluate the impact of symptom screening at a population level.

We used linked administrative healthcare data to create a propensity matched cohort of individuals who have or have not reported symptoms on ESAS and examine the impact of ESAS exposure on overall survival. The methods allow us to better control for measured and unmeasured confounding variables and facilitate a comparison of the two groups using retrospective observational data. We hypothesized that exposure to ESAS would be associated with improved overall survival.

## 2 METHODS

### 2.1 Study setting and population

To evaluate the effect of ESAS screening on overall survival, a retrospective population‐wide exposure‐matched study was conducted. We identified all patients with diagnosed with cancer from January, 2007 through December, 2015 from the Ontario Cancer Registry in Ontario, Canada. Patients had to be age 18 or older at the time of diagnosis. Individuals with a previous history of cancer or with multiple cancers were excluded, as were patients with invalid or missing information on demographics. Institutional ethics approval was obtained prior to commencing.

### 2.2 Data sources

We linked relevant health administrative databases held at the ICES using unique encoded identifiers to create the cohort. The Ontario Cancer Registry^12^, ^13^ identified all incident cancers in Ontario while the Symptom Management Reporting Database identified patients who used ESAS. The Registered Persons Database^14^ provided demographic information on all Ontarians including date of death from any cause and their eligibility to receive care under Ontario Health Insurance Plan (OHIP). The OHIP database recorded all visits to physicians and the Canadian Institute for Health Information's National Ambulatory Care Reporting System^15^ recorded visits to emergency rooms and cancer centers. All hospitalizations or same day surgeries were obtained from the Discharge Abstract and Same Day Surgery databases. All activities during regional cancer center visits were obtained from Activity Level Reporting database.

### 2.3 Exposure and matching

Exposed patients were those completing at least one ESAS assessment during the study, while unexposed patients had never used ESAS. Every cancer centre had a system in place to capture patients’ symptoms via a touch screen kiosk, hence the ability to capture symptoms at every visit exists as a matter of routine; however, the strength of the implementation would affect the proportion of patients who actually complete ESAS.^10^ Examples of reasons for non‐exposure would include patients being roomed immediately after check in, patient preference, absent volunteers (in an implementation that relies on volunteers), poor staff engagement or staff turnover.

The index date for the exposed patients was the first ESAS assessment date after the cancer diagnosis. For each patient exposed to ESAS, we aimed to find 1 unexposed patient via both hard and propensity score matching. Patients were hard matched on year of birth (±2 years), date of cancer diagnosis (±1 year), cancer type and sex. Logistic regression was used to calculate the propensity score of completing an ESAS assessment. The model included patient characteristics (age, sex, neighborhood income quintile, urban/rural residence, region), cancer characteristics (type, stage, year of diagnosis), treatments within 6 months of diagnosis (chemotherapy, radiation and surgery), various measures of comorbid conditions in the past 2 years (total Charlson score,^16^, ^17^ total Aggregated Diagnosis Groups score and Resource Utilization Bands score from John Hopkins Adjusted Clinical Groups System^18^), and number of visits to the emergency department in the 2 years prior to cancer diagnosis. Exposed and unexposed patients were matched on a caliper width of 0.2 standard deviations of the log odds of the estimated propensity score.

**Figure 1:**
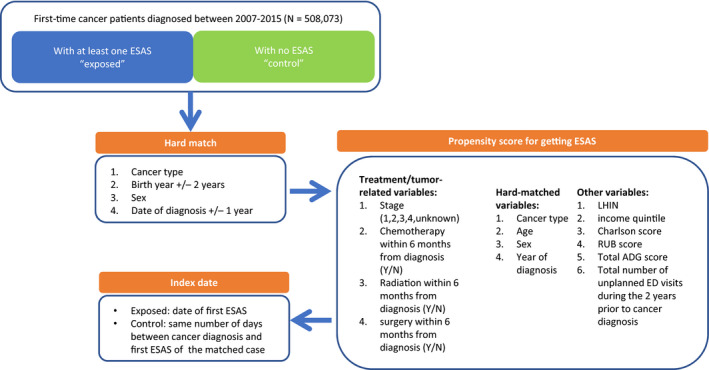
Creation of matched pairs and definition of index date

Upon completion of matching, a dummy index date were assigned to each unexposed patient such that the gap time (in days) between their diagnosis date and dummy index date was the same as the gap time between the corresponding exposed patient's diagnosis date and first ESAS date (Figure 1). Patients were followed from their index date until death, diagnosis of a new cancer, 5‐year observation mark, or the end of study at December 31, 2015, whichever came first. The 5‐year time point is often used for the evaluation of survival.

### 2.4 Phase of care (defined in order to be incorporated as a time‐varying covariate)

The study period between index date and end of observation for each patient was segmented into one of three cancer management‐related phases: initial, continuing, or palliative care. For patients diagnosed with stage I‐III cancer, the initial phase included first 12 months after diagnosis. The palliative care phase started when a patient (a) was initially diagnosed with stage IV or subsequently found to have metastases; (b) initiated chemotherapy or radiation after 12 months of cancer diagnosis; (c) re‐started chemotherapy or radiation after 3‐month interval (d) started chemotherapy or radiation with a palliative intent; or (e) received palliative care services. The continuing phase which includes ongoing surveillance and routine follow up care was assigned to the time between initial and palliative care phases. Not all patients were assigned to every phase of care. For example, a patient who was diagnosed with a stage IV cancer would have only been assigned to the palliative care phase. For each patient, as we move along their observation timeline, it is important to note that this phase of care definition did not require looking forward or ahead into time to determine one's current phase of care status; it's definition only depended on the occurrences of events up to the given point in time, which thus makes it a suitable time‐varying covariate.^19^

### 2.5 Statistical analysis

Baseline characteristics of matched exposed and unexposed patients were compared using standardized differences. A difference larger than 10% was felt to represent meaningful imbalance in the given characteristic between the exposed and unexposed group.^20^ Kaplan‐Meier method and log‐rank test were used to compare the unadjusted probabilities of survival between the two groups. Multivariable Cox proportional hazards model was used to evaluate the impact of ESAS assessment on survival. A robust sandwich variance estimator was used in the model to account for correlation within matched pairs. Since the matching process balanced many baseline factors between the exposed and unexposed, the multivariable model only adjusted for the following additional measures: number of visits to a radiation or medical oncologist between cancer diagnosis and index date was incorporated as a fixed covariate measured at index; number of visits to a family physician or radiation/medical oncologist between the index date and end of follow‐up was captured as counter time‐varying covariate; and experiencing surgery after diagnosis was captured as a binary time‐varying covariate that turned “on” once surgery was received.

Phase of care was defined and included as a 3‐level categorical time‐dependent covariate. The category a patient belonged to depended on the phase of care they were in at that specific point in time.

In a separate multivariable Cox model, we also incorporated an interaction between ESAS exposure and phase of care to assess the variation in the impact of ESAS on survival during each phase of care. Indicators for chemotherapy and radiation were not included in the model as they were part of phase of care definition.

All statistical analyses were done in SAS 14.2 (SAS Institute, Inc, Cary, NC).

## 3 RESULTS

### 3.1 Baseline Characteristics

Of 508 073 patients who were diagnosed with a cancer, 213 887 had at least one ESAS assessment during the study period. We successfully matched 128,893 (60.3%) patients with ESAS exposure to 128 893 patients without ESAS exposure. Before matching, several differences were present between exposed and unexposed patients: exposed patients were more likely to be younger, women, living in areas of higher income quintile, diagnosed with breast, gynecological, or hematology cancer, not missing cancer stage, in early stages of cancer, having fewer comorbid conditions, having less healthcare services in the past and more cancer treatments. After matching, baseline characteristics of the matched patients were similar between the two groups (Table 1). The median follow up was 1.4 years. The median time spent in the initial, continuing or palliative phase for the group without ESAS was 0.5 years (IQR 0.2‐0.8), 1.8 (0.8‐3.8), and 0.3 (0.09‐1.0), respectively. For those with ESAS the median time spent in the initial, continuing or palliative phase was 0.6 years (0.3‐0.8), 1.6 (0.7‐3.7) and 0.8 (0.3‐1.7), respectively. During the study period, those exposed had a median of 3 (IQR 2‐8) ESAS assessments. Seventy‐five percent of patients had more than one ESAS. 98.5% completed all nine symptoms.

**Table 1:** Patient characteristics by exposure status (N = 128 893 pairs)

Variable	Value	ESAS = No (N = 128 893)	ESAS = Yes (N = 128 893)	Standardized difference
Age at cancer diagnosis	Mean ± SD	64.28 ± 12.97	64.35 ± 12.97	0.01
Sex	Female	61 549 (47.8%)	61 549 (47.8%)	Matched Variable
	Male	67 344 (52.2%)	67 344 (52.2%)	
Cancer type	Brain	1439 (1.1%)	1439 (1.1%)	Matched Variable
	Breast	18 642 (14.5%)	18 642 (14.5%)	
	Colorectal	14 240 (11.0%)	14 240 (11.0%)	
	Gynaecological	9657 (7.5%)	9657 (7.5%)	
	Head and Neck	3822 (3.0%)	3822 (3.0%)	
	Hematology	17 213 (13.4%)	17 213 (13.4%)	
	Lung	16 537 (12.8%)	16 537 (12.8%)	
	Melanoma	4568 (3.5%)	4568 (3.5%)	
	Non‐melanoma	340 (0.3%)	340 (0.3%)	
	Prostate	22 418 (17.4%)	22 418 (17.4%)	
	Thyroid	2215 (1.7%)	2215 (1.7%)	
	Other Gastrointestinal	9626 (7.5%)	9626 (7.5%)	
	Other Genitourinary	5743 (4.5%)	5743 (4.5%)	
	Other	1650 (1.3%)	1650 (1.3%)	
	Unknown primary	783 (0.6%)	783 (0.6%)	
Cancer stage	0	381 (0.3%)	316 (0.2%)	0.01
	I	29 923 (23.2%)	27 164 (21.1%)	0.05
	II	29 854 (23.2%)	28 640 (22.2%)	0.02
	III	16 612 (12.9%)	17 040 (13.2%)	0.01
	IV	16 417 (12.7%)	20 349 (15.8%)	0.09
	Unknown	35 706 (27.7%)	35 384 (27.5%)	0.01
Neighbourhood Income Quintile	Lowest	22 936 (17.8%)	23 220 (18.0%)	0.01
	Next to lowest	25 542 (19.8%)	25 631 (19.9%)	0
	Middle	25 416 (19.7%)	25 521 (19.8%)	0
	Next to highest	27 191 (21.1%)	27 042 (21.0%)	0
	Highest	27 808 (21.6%)	27 479 (21.3%)	0.01
Number of inpatient admission in 2 years prior to diagnosis	0	107 487 (83.4%)	107 224 (83.2%)	0.01
	1	15 609 (12.1%)	15 843 (12.3%)	0.01
	2	3811 (3.0%)	3847 (3.0%)	0
	3+	1986 (1.5%)	1979 (1.5%)	0
Number of unplanned visits to emergency department in 2 years prior to diagnosis	0	64 944 (50.4%)	64 776 (50.3%)	0
	1	30 685 (23.8%)	30 072 (23.3%)	0.01
	2	14 643 (11.4%)	14 694 (11.4%)	0
	3+	18 621 (14.4%)	19 351 (15.0%)	0.02
Charlson score (comorbidities in 2 years prior to diagnosis)	0	113 986 (88.4%)	113 580 (88.1%)	0.01
	1	7459 (5.8%)	7405 (5.7%)	0
	2	4072 (3.2%)	4349 (3.4%)	0.01
	3+	3376 (2.6%)	3559 (2.8%)	0.01
Surgery within 6 months after diagnosis	No	60 068 (46.6%)	64 089 (49.7%)	0.06
	Yes	68 825 (53.4%)	64 804 (50.3%)	0.06
Chemotherapy within 6 months after diagnosis	No	95 780 (74.3%)	94 538 (73.3%)	0.02
	Yes	33 113 (25.7%)	34 355 (26.7%)	0.02
Radiation within 6 months after diagnosis	No	99 235 (77.0%)	98 293 (76.3%)	0.02
	Yes	29 658 (23.0%)	30 600 (23.7%)	0.02
Number of years between cancer diagnosis and index date	Mean ± SD	1.1 (1.6)	1.1 (1.6)	Matched Variable
	Median (IQR)	0.3 (0.1‐1.5)	0.3 (0.1‐1.5)	

### 3.2 Overall survival

**Figure 2:**
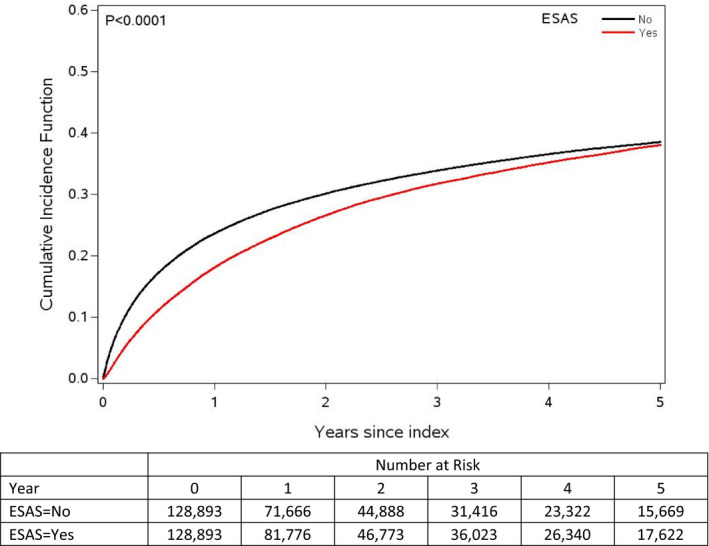
Cumulative incidence function of death for patients exposed and unexposed to ESAS

The probability of survival within the first five years was higher among those exposed to ESAS compared to those who were not (81.9% vs. 76.4% at 1 year, 68.3% vs. 66.1% at 3 years, 61.9% vs. 61.4% at 5 years, *P*‐value < .0001, Figure 2).

In the multivariable Cox regression model, ESAS assessment was significantly associated with a decreased mortality risk (HR: 0.48, 95% CI: 0.47‐0.49, Table 2). As expected, in the base model, initial or palliative phase of care are associated with a higher hazard of death when compared to continuing phase.

**Table 2:** Results of univariable and multivariable Cox proportional hazards model with robust variance estimator examining time to death

Variables	Comparison	Univariable Hazard ratio (95% Confidence Interval)	Multivariable Hazard ratio (95% Confidence Interval)
Exposure to ESAS	Yes vs No	0.85 (0.84, 0.86)	0.48 (0.47, 0.49)
Surgery after diagnosis (time‐dependent)	Yes vs No	0.364 (0.36, 0.37)	0.58 (0.57, 0.59)
Number of clinic visits to radiation/ medical oncologist from diagnosis to index		0.992 (0.99, 0.99)	0.967 (0.96, 0.97)
Number of clinic visits to family or radiation/medical oncologist after index		1.031 (1.03, 1.03)	1.025 (1.023, 1.028)
Phase of care	Initial vs Continuing	1.52 (1.45, 1.59)	1.40 (1.34, 1.47)
	Palliative vs Continuing	27.76 (26.91, 28.64)	28.68 (27.76, 29.63)

**Figure 3:**
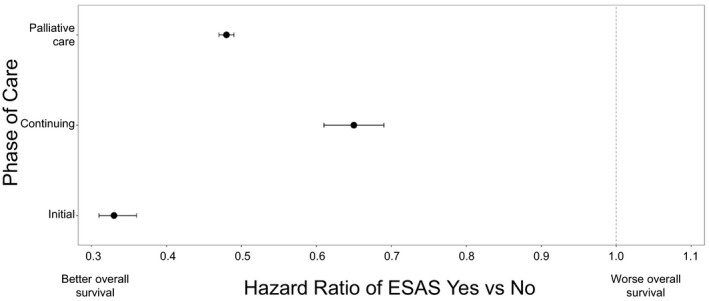
The impact of ESAS assessment on overall survival by phase of care

We added an interaction term between ESAS assessment and phase of care in a separate model to evaluate whether the impact of ESAS on mortality varied between different phases of care. A statistically significant association between ESAS assessment and reduced mortality was seen across all phases and the reduction in risk was highest in the initial phase (HR: 0.33 95% CI: 0.31‐0.36), followed by the palliative phase (HR: 0.48 95% CI: 0.47‐0.49) followed by the continuing phase (HR: 0.67 95% CI: 0.63‐0.71) (Figure 3). Addition of this interaction term did not change the estimated coefficients of the other covariates in the model. Surgery within 6 months of diagnosis and an increased number of clinic visits to radiation or medical oncologists between diagnosis and index date were associated with a reduced mortality risk whereas an increased number of clinic visits to a family physician, radiation or medical oncologist after index was associated with an increased mortality risk. In a separate model as a sensitivity analysis, we examined the effect of exposure to ESAS on overall survival without limiting the follow up to 5 years and the main effect was unchanged.

The R‐square value for the univariable model is 0.0019, for the multivariable model is 0.4261 and for the multivariable model with the interaction is 0.4265. The R‐square value improves substantially with the main multivariable model and a small amount more with the addition of the interaction term.

## 4 DISCUSSION

We used administrative health care data to create a matched cohort of cancer patients who were and were not exposed to ESAS. We observed that those exposed to ESAS lived longer than those who were not. We further observed that the association between ESAS exposure and survival was strongest in the initial phase and in the palliative phase. This finding provides further real world evidence of the impact of routine use of PROs in clinical care. Prior work has demonstrated those exposed to ESAS are less likely to go to the emergency department or be hospitalized and are more likely to be referred to palliative care.^21^, ^22^

Our results are consistent with randomized studies that have evaluated overall survival as an endpoint. Namely, Basch et al^7^ found that routine symptom monitoring in cancer patients with solid tumors on chemotherapy had a hazard of 0.83 (95% CI 0.70‐0.99) compared to those who did not have routine symptom monitoring. Denis et al^8^ evaluated the impact of routine symptom monitoring from home in patients with lung cancer using an algorithmic approach to recall patients based on predefined criteria for symptom severity and worsening. They reported improved overall survival with weekly symptom monitoring compared with three monthly clinic visits and more frequent diagnostic imaging (HR 0.59, 95% CI: 0.37‐0.96). Both of these studies asked questions about symptoms commonly experienced by cancer patients and have overlapping items with ESAS, such as pain, appetite, shortness of breath, fatigue, nausea, or depression. This manuscript adds to the existing literature because it evaluates the impact of symptom screening in the real world context. In randomized studies where eligibility criteria are strict and interventions tightly defined, results may have limited generalizability. These limitations do not exist with population based data where the particulars of how symptom screening happens are not controlled.

We observed the strongest association between ESAS exposure and survival when it occurred in the first year after diagnosis (when most receive treatment) or after the cancer recurred. This suggests that the impact is largest when patients are sicker. This is consistent with the two randomized trials^7^, ^8^ that identified survival benefit, which also targeted sicker or metastatic patients. The possible mechanisms leading to benefit include earlier symptom identification or more comprehensive symptom identification whose management directly benefits the patient; alternatively, improvements in symptom management may also have allowed patients to stay on chemotherapy longer. In other work (submitted) we demonstrate a small increase in palliative care referrals for those exposed to ESAS. Early palliative care has also been shown to improve survival.^23^

For patients who are well and attending routine surveillance visits the routine use of ESAS was associated with smaller survival improvement. There may be less opportunity to improve outcome for patients in the follow up or surveillance phase of care. Their symptom burden based on ESAS may be less and as such the impact of symptom management may be minimal. Measures that focus on long term toxicities specific to cancer type or treatment and that focus on survivorship issues may have more of an impact in this group. The quality of life or other end points may also be more relevant for this group.

The strengths of this paper are the comprehensive nature of the data used. We were able to create a matched cohort of 128,893 pairs of patients. We hard matched on 4 variables and then matched further on a propensity score created with 14 variables. We included common oncologic prognostic variables such as age, sex, cancer type, and stage. This would be the highest quality comparison of two groups that could be made with observational data. To the extent possible, extensive matching methods have mitigated biases inherent to observational data. The very small differences that existed (eg stage or surgery) favored the unexposed group.

However, limitations include that the data may be missing important clinical prognostic information which may be different between the two groups. As ESAS measurement is considered routine in Ontario, there may be other unmeasured confounders between patients who do and do not avail themselves of it. The direction of this bias is difficult to estimate. For example, patients with multiple symptoms might be keen to report their symptoms and very likely to go to the kiosk; alternatively, they might feel too unwell to go to the kiosk and report their symptoms. We note that the direction of the estimate of ESAS association with survival remained consistent between univariable (HR 0.85) and multivariable (HR 0.48) analyses, implying the message is the same even after adjusting for obvious confounders. ESAS completion may be associated with other factors such as increased health literacy or ability to self‐manage symptoms. Finally, the algorithm to assign phases of care has not been validated with a chart review. It is possible for example, that patients receiving curative intent therapy also received palliative care services for symptom management. Our algorithm would incorrectly label that phase of care as palliative. This limitation would not have any impact on the finding of the main model, and is unlikely to occur frequently enough to change the direction of the interaction findings.

This paper provides real world evidence of the impact of routine patient reported outcome use in clinic. Improvements in survival are seen, in particular for patients in the first year after diagnosis or after recurrence. This adds to the body of evidence to support the important role of routine standardized symptom assessment. Future studies should include survival as an endpoint to further develop this evidentiary base. Generic measures that include common symptoms seem to be adequate for this outcome. Disease specific measures and other outcomes may be more relevant for patients who are well and in the surveillance part of their journey.

## PRIOR PRESENTATION

This work was presented in abstract form at the 2019 annual American Society for Clinical Oncology (ASCO) meeting.

## ETHICS

Institutional ethics approval was obtained prior to commencing.

## Conflict of Interest

LB has received an honorarium from Genentech. DH reports personal fees from CareVive Technology Company. All other authors have nothing to disclose.

## Author Contributions

LB, RS, HS, NM, DH, and CE contributed to concept/design. LB, RS, HS, NM, DH, and CE contributed to funding. LB and RS contributed to draft manuscript. All authors contributed to analysis and interpretation and approved the final manuscirpt. LB is the Guarantor of the study.
